# Placebo response rates and potential modifiers in double-blind randomized controlled trials of second and newer generation antidepressants for major depressive disorder in children and adolescents: a systematic review and meta-regression analysis

**DOI:** 10.1007/s00787-018-1244-7

**Published:** 2018-12-08

**Authors:** Ramona Meister, Mariam Abbas, Jochen Antel, Triinu Peters, Yiqi Pan, Ulrike Bingel, Yvonne Nestoriuc, Johannes Hebebrand

**Affiliations:** 1 Department of Medical Psychology, University Medical Center Hamburg-Eppendorf, Martinistraße 52, 20246 Hamburg, Germany; 2 Department of Child and Adolescent Psychiatry, University Hospital Essen, University of Duisburg-Essen, Essen, Germany; 3 Department of Psychosomatic Medicine and Psychotherapy, University Medical Center Hamburg-Eppendorf, Hamburg, Germany; 4 Department of Neurology, University Hospital Essen, University of Duisburg-Essen, Essen, Germany; 5 Clinical Psychology, Helmut-Schmidt-University, University of the Federal Armed Forces Hamburg, Hamburg, Germany

**Keywords:** Major depressive disorder, Placebo response rates, Modifiers of placebo effect, Meta-analysis, Children and adolescents

## Abstract

Children and adolescents with major depressive disorder (MDD) appear to be more responsive to placebo than adults in randomized placebo-controlled trials (RCTs) of second and newer generation antidepressants (SNG-AD). Previous meta-analyses obtained conflicting results regarding modifiers. We aimed to conduct a meta-analytical evaluation of placebo response rates based on both clinician-rating and self-rating scales. Based on the most recent and comprehensive study on adult data, we tested whether the placebo response rates in children and adolescents with MDD also increase with study duration and number of study sites. We searched systematically for published RCTs of SNG-AD in children and/or adolescents (last update: September 2017) in public domain electronic databases and additionally for documented studies in clinical trial databases. The log-transformed odds of placebo response were meta-analytically analyzed. The primary and secondary outcomes were placebo response rates at the end of treatment based on clinician-rating and self-rating scales, respectively. To examine the impact of study duration and number of study sites on placebo response rates, we performed simple meta-regression analyses. We selected other potential modifiers of placebo response based on significance in at least one previous pediatric meta-analysis and on theoretical considerations to perform explorative analyses. We applied sensitivity analyses with placebo response rates closest to week 8 to compare our data with those reported for adults. We identified 24 placebo-controlled trials (2229 patients in the placebo arms). The clinician-rated placebo response rates ranged from 22 to 62% with a pooled response rate of 45% (95% CI 41–50%). The number of study sites was a significant modifier in the simple meta-regression analysis [odds ratio (OR) 1.01, 95% CI 1.01–1.02, *p* = 0.0003, *k* = 24) with more study sites linked to a higher placebo response. Study duration was not significantly associated with the placebo response rate. The explorative simple analyses revealed that publication year may be an additional modifier. However, in the explorative multivariable analysis including the number of study sites and the publication year only the number of study sites reached a *p* value ≤ 0.05. The self-rated placebo response rates ranged from 1 to 68% with a pooled response rate of 26% (95% CI 10–54%) (*k* = 6; *n* = 396). This meta-analysis confirms a high pooled placebo response rate in children and adolescents based on clinician ratings, which exceeds that observed in the most recent meta-analysis of placebo effects in adults (36%; 95% CI 35–37%) published in 2016. However, and similar to findings in adults, the pooled response rates based on self-ratings were substantially lower. In accordance with previous meta-analyses, we corroborated the number of study sites as significant modifier. In comparison to the recent adult meta-analysis, the substantially lower number of pediatric studies entails a reduced power to detect modifiers. Future studies should provide more precise and homogenous information to support discovery of potential modifiers and consider no-treatment—if ethically permissible—to allow differentiation between placebo and spontaneous remission rates. If these differ, practicing clinicians should facilitate placebo effects as an addition to the *verum* effect to maximize benefits. Further research is required to explain the discrepant response rates between clinician and self-ratings.

**Electronic supplementary material:**

The online version of this article (10.1007/s00787-018-1244-7) contains supplementary material, which is available to authorized users.

## Introduction

Depressive disorders are one of the most common life-shortening diseases worldwide [1, 2] and may severely burden patients, their family members and employers [3], as well as the public health systems [4]. The medical need for efficient, patient-centered, and cost-effective treatment [5] is large, especially for children and adolescents [6, 7]. The 1-year prevalence rates over the whole age range for all mental disorders and MDD in Europe were 40% and 6.9% in 2011, respectively [8]. The estimated point prevalence rates for MDD were 2.8% in children aged 6–12 years and 5.6% in adolescents according to a US study [9]. The incidence in juveniles rises steeply, especially in girls after puberty [10]. Symptoms of mental disorders frequently arise in childhood or adolescence and persist into adulthood [9, 11–16]. About half of the cases diagnosed in adulthood with a mental disorder date their first symptoms back to early adolescence [17].

The current armamentarium for treating depressive disorders in children and adolescents [16] is meager and represents a huge medical need. Most clinical guidelines [18–20] recommend psychological interventions as first-line treatment for children and adolescents. However, antidepressants are widely used with increasing prescription rates [21]. In many cases, antidepressants are prescribed off-label since an approval for children and adolescents is lacking for most antidepressants. A recent network meta-analysis by Cipriani and colleagues [22] provided some evidence that among 14 antidepressant treatments, only fluoxetine is more effective than placebo in reducing depressive symptoms in children and adolescents with MDD.

In the most recent adult meta-regression analysis, the average clinician-rated placebo response rate was 36% (95% CI 35–37%) with a range of 0–70% [23], thus revealing a high proportion of non-pharmaceutically mediated effects in clinical trials of antidepressants. In children and adolescents, Cohen et al. [24] observed an even higher average placebo response rate of 49.6% with a wide range of 17–90% in a study including both tricyclic antidepressants and SNG-AD.

Given the large placebo effects, it is warranted to increment our understanding of modifiers which increase or decrease placebo response rates [25, 26]. The main underlying and most established mechanisms of individual placebo effects with the highest empirical evidence are treatment expectation, behavioral conditioning, and the quality of the patient-physician relationship [25]. Earlier studies based on adults suffering from MDD found a higher placebo response rate with increasing publication year [27, 28], and longer study duration [28]. However, the most recent systematic meta-analysis of placebo response rates in studies of first, second and newer generation antidepressants for acute treatment of adult MDD revealed that the average placebo response rate-defined as ≥ 50% reduction in depression severity scores on a standardized clinician-rating scale-has remained constant since the year 1991 in a multivariable model [23]. Moreover, this large scale meta-analysis [23] found that, of all analyzed potential modifiers, placebo response rates increased with longer study duration and with a larger number of study sites.

It is so far unknown whether the results on placebo response rates and their modifiers in adult MDD RCTs can be transferred to children and adolescents and vice versa. Investigators [29, 30] have argued that there is little reason to extrapolate adult data to children because of the neurodevelopmental and psychological differences. These differences also include potential modifiers. For example, adults have more likely experienced two or more depressive episodes, thus entailing that the total duration of the disorder and chronic courses more frequently affect adults than children or adolescents. Differences in the placebo response rates are also mirrored in the two recent meta-analyses of Cipriani et al. [31, 32] which revealed that all analyzed antidepressants were more efficacious than placebo in adults with MDD [32] in contrast to the results in children and adolescents, for whom only fluoxetine was cautiously viewed as efficacious [31]. These differences suggest that the results on placebo response rates and their modifiers obtained in adult studies cannot readily be extrapolated to children and adolescents, thus warranting further pediatric analyses.

To our knowledge, seven systematic reviews and meta-regression analyses [22, 24, 29, 33–36] examined placebo response rates in children and adolescents with MDD (some also addressed additional disorders). All focused on clinician based response rates; self-rated placebo response rates have not been meta-analyzed. The most comparable study [33] to our own focused also on both placebo response rates and their modifiers in youth with MDD; it is now nearly a decade old and included exactly half (*k* = 12; *n* = 2862) of the studies that were now available for analysis. The most recent meta-analysis of Locher and coworkers [35] covered 17 of the 24 studies included in our current analysis. Locher and colleagues also analyzed the placebo response rate in studies of various psychiatric disorders including MDD defined as the mean change scores of preanalyses vs. postanalyses in the placebo group. However, modifiers were only analyzed for the drug-placebo differences [35]. In accordance with Bridge et al. [33], we focused on placebo response rates and their modifiers per se. In addition to SSRI and SNRI, we included the recent studies on SNG-ADs including the serotonin antagonist and reuptake inhibitor (SARI) nefazodone (*k* = 2; [37, 38]) and the noradrenergic and specific serotonergic antidepressant (NaSSA) remeron (*k* = 2; [39, 40]).

We therefore aimed to meta-analytically investigate placebo response rates for SNG-antidepressants in studies on children and adolescents with MDD using a methodological approach similar to that of Bridge and coworkers [33]. Moreover, we aimed to follow up on the most recent and comprehensive study on adult data [23] and test whether the clinician-rated placebo response rate in children and adolescents with MDD also increases with increasing study duration and number of study sites. The effect of both modifiers had also been investigated by Bridge et al. [33]: whereas the number of study sites proved to be a significant modifier, this was not the case for study duration (*r* = 0.13, 95%CI − 0.67 to 0.80; *p* > 0.05, *k* = 12).

In addition, we explored three other previously identified direct modifiers of placebo response (in contrast to modifiers of drug-placebo differences [34, 35]) in meta-regression analyses of studies on children and adolescents with MDD): publication year [33], mean baseline severity [33], and sample size [33].

Finally, we assessed seven additional modifiers due to the following considerations: (1) because of the significance of study duration in the adult meta-regression conducted by Furukawa et al. [23] we also assessed the effect of a run-in phase as a time component as well as risk of bias despite previous negative results [33, 34], (2) concomitant psychotherapy was analyzed as an additional potential modifier because psychotherapy may influence expectations towards the efficacy of drug/placebo treatment, (3) based on an anonymous reviewer’s comment we analyzed the proportion of females, funding source, mean age and chance to receive placebo (in two- vs. more armed studies).

## Materials and methods

We conducted the systematic review and meta-regression analysis in accordance with current guidelines [41] and prepared the manuscript in accordance with the Preferred Reporting Items for Systematic Reviews and Meta-Analysis (PRISMA) statement [42].

### Eligibility criteria

We included all double-blind RCTs comparing SNG-AD (defined as all antidepressants that were introduced since the 1970s as follow-ups of tricyclic antidepressants and monoamine oxidase inhibitors; for details see Table 1) with placebo (as oral monotherapy) in the acute treatment of children and adolescents (age range 6–18) of both sexes with a primary diagnosis of MDD. For eligibility, the diagnosis had to rely on standardized diagnostic criteria, i.e., according to the actual DSM or ICD versions at the time of publication of the respective study. In line with the adult meta-analysis of placebo response rates [23], we only included data from studies pertaining to acute treatment of MDD. However, our search criteria did not reveal any trial with duration of less than 6 or more than 12 weeks; this time span is in accordance with the definition of acute treatment of Furukawa et al. [23]. Trials involving patients with comorbid, non-affective psychiatric disorders (e.g., “comorbid alcohol and cannabis use disorders”; [43]), and trials which did not prohibit concomitant psychotherapeutic treatments on top of the study treatment were not excluded to increase external validity of results. However, studies which included psychotherapy as part of the treatment and/or placebo arm were excluded. Our primary outcome was the placebo response rate at the end of the intervention as assessed with a clinician-rating scale. Our secondary outcome was the placebo response rate at the end of intervention measured using a self-rating scale.

**Table 1:** Characteristics of the 24 eligible studies

Study	*N*	Diagnostic criteria	Relevant study arms	C.P.	Mean age (range)	% female	% White (W) or caucasian (C)	Treatment duration (weeks)	Funder	Baseline score severity (clinician rated)	Baseline score severity (self-rated)	PBO run-in	PT outside the study	*N* sites	Country	Response criteria
Atkinson et al. [44]	337	DSM-IV-TR for MDD without psychotic features	Duloxetine (60–120 mg), fluoxetine (20–40 mg), placebo	1/3	13.2 (7–17)	52	78 W	10	Yes	59.4 (CDRS) moderate	NR	No	Not mentioned as exclusion criterion	65	US, FI, FR, DE, SK, EE, RU, UA, ZA	CDRS-R reduction ≥ 50%
Berard et al. [45]	286	DSM-IV for unipolar MDD	Paroxetine (20–40 mg), placebo	1/2	15.6 (12–18)	67	68 C	12	Yes	25.9 (MADRS) moderate	22.8 (BDI) moderate	Yes	Routine short-term supportive PT or family supportive therapy allowed	33	BE, IT, ES, GB, NL, CA, ZA, AE, AR, MX	MADRS reduction ≥ 50% CGI-I ≤ 2
US Food and Drug Administration [37, 46]	284	Non-psychotic MDD	Nefazodon (100–300 mg), Nefazodon (200–600 mg), placebo	1/3	12 (7–17)	≈ 50	73 C	8	Yes	60.1 (CDRS) moderate	NR	NR → No	NR	28	US	NR; estimated by means of the reported baseline scores, endpoint scores and its standard deviation
Lilly [47]	40	DSM-III for MDD	Fluoxetine (20–60 mg), placebo	1/2	15.6 (12–17)	55	100 C	6	Yes	21.9 (HAMD) moderate	113.1 (SCL-58) moderate	Yes	NR	1	NR	NR; estimated by means of the reported baseline scores, endpoint scores and its standard deviation
Emslie et al. [48]	96	DSM-III-R for non-psychotic MDD	Fluoxetine (20 mg), placebo	1/2	12.4 (7–17)	46	79 W	8	No	58.1 (CDRS) moderate	15.6 (BDI) mild	Yes	Not mentioned as exclusion criterion	NR	NR	CGI-I ≤ 2
Emslie et al. [49]	219	DSM-IV for non-psychotic MDD	Fluoxetine (10–20 mg), placebo	1/2	12.7 (8–18)	49	82 W	9	Yes	56.1 (CDRS) moderate	NR	Yes	Supportive PT or family supportive therapy allowed	15	US	CDRS-R reduction ≥ 30% CDRS-R reduction ≥ 50%
US Food and Drug Administration [37, 46]\Emslie et al. [38]	206	DSM-IV for MDD	Nefazodone (100–400 mg), placebo	1/2	15 (12–18)	60	78 C	8	Yes	NR	NR	No	NR	15	US	CGI-I ≤ 2
Emslie et al. [50]	206	DSM-IV for non-psychotic MDD	Paroxetine (10–50 mg), placebo	1/2	12 (7–17)	47	79 W	8	Yes	61.7 (CDRS) Severe	17.9 (KADS, adolescents only) moderate	No	PT prohibited	41	US., CA	CGI-I ≤ 2
Emslie et al. [51]	367 (com)	DSM-IV for non-psychotic MDD	Venlafaxine (37.5–225 mg), placebo	1/2	12.2 (7–17) (com)	45 (com)	NR	8	Yes	54.4 (CDRS) moderate		Yes	Not mentioned as exclusion criterion	14	US	CDRS-R reduction ≥ 35% HAMD/MADRS reduction ≥ 50% CGI-I ≤ 2
Emslie et al. [51]	367 (com)	DSM-IV for non-psychotic MDD	Venlafaxine (37.5–225 mg), placebo	1/2	12.2 (7–17) (com)	45 (com)	NR	8	Yes	57.3 (CDRS) moderate		Yes	Not mentioned as exclusion criterion	37	US	CDRS-R reduction ≥ 35% HAMD/MADRS reduction ≥ 50% CGI-I ≤ 2
Emslie et al. [52]	316	DSM-IV for non-psychotic MDD	Escitalopram (10–20 mg), placebo	1/2	14.6 (12–17)	59	76 W	8	Yes	56.8 (CDRS) moderate	NR	Yes	PT prohibited	40	US	CDRS-R reduction ≥ 40% CDRS-R reduction ≥ 50% CGI-I ≤ 2
Emslie et al. [53]	463	DSM-IV-R without psychotic features	Duloxetine (60 mg), Duloxetine (30 mg), Fluoxetine (20 mg), placebo	1/4	13 (7–17)	51	53 W	10	Yes	58.8 (CDRS) moderate	NR	No	Not mentioned as exclusion criterion	60	US, CA, MX, AR	CDRS-R reduction ≥ 50%
Findling et al. [43]	34	DSM-IV for non-psychotic MDD or depressive disorder with a comorbid substance-related disorder	Fluoxetine (10–20 mg), placebo	1/2	16.5 (12-17)	15	73 W	8	Yes	53.4 (CDRS) moderate	15.2 (BDI) mild	No	PT allowed (with limited intensity)	1	US	CDRS-R reduction ≥ 30% CGI-I ≤ 2
GSK [54]	56	DSM-IV-TR without psychotic features	Paroxetine (10–40 mg), placebo	1/2	14.6 (7–17)	61	NR	8	Yes	NR	NR	Yes	Not mentioned as exclusion criterion	33	JP	CGI-I ≤ 2
Keller et al. [55]	275	DSM-IV for MDD	Paroxetine (20–40 mg), Imipramine* (200–300 mg), placebo	1/3	14.9 (12–18)	62	84	8	Yes	18.7 (HAMD) moderate	NR	No	PT prohibited	12	US, CA	HAM-D ≤ 8 or ≥ 50% Reduction CGI-I ≤ 2
March et al. [56]	439	DSM-IV for MDD	Fluoxetine (10–40 mg) + CBT*, Fluoxetine alone (10–40 mg), CBT alone*, placebo	1/4	14.6 (12–17)	54	74 W	12	No	60.1 (CDRS) moderate	79.2 (RADS) moderate	No	PT prohibited	13	US	CGI-I ≤ 2
US Food and Drug Administration [39]/Cheung et al. [40]	126	DSM-IV for non-psychotic MDD	Remeron (15–45 mg), placebo	1/2	12.3 (7–17)	51	83 C	8	Yes	NR	NR	NR → No	NR	17	NR	CGI-I ≤ 2
US Food and Drug Administration [39]/Cheung et al. [40]	133	DSM-IV for non-psychotic MDD	Remeron (15–45 mg), placebo	1/2	12 (7–17)	53	78 C	8	Yes	NR	NR	NR → No	NR	15	NR	CGI-I ≤ 2
Von Knorring et al. [57]	244	DSM-IV for non-psychotic MDD	Citalopram (20–40 mg), placebo	1/2	16 (13–18)	NR	NR	12	Yes	30 (MADRS) moderate	NR	No	PT allowed	31	Eur.	MADRS reduction ≥ 50%
Wagner et al. [58]	376	DSM-IV for non-psychotic MDD	Sertraline (50–200 mg), placebo	1/2	NR (6–17)	51	70 W	10	Yes	64.4 (CDRS) Severe	NR	No	PT allowed	53	US, IN, CA, CR, MX	CDRS-R reduction ≥ 40% CGI-I ≤ 2
Wagner et al. [59]	178	DSM-IV for non-psychotic MDD	Citalopram (20–40 mg), placebo	1/2	12.1 (7–17)	53	77 C	8	Yes	58.3 (CDRS) moderate	NR	Yes	PT prohibited	21	US	CDRS-R ≤ 28 CGI-I ≤ 2
Wagner et al. [60]	268	DSM-IV for non-psychotic MDD	Escitalopram (10–20 mg), placebo	1/2	12.3 (6–17)	52	71 W	8	Yes	55.6 (CDRS) moderate	NR	Yes	PT prohibited	25	US	CDRS-R ≤ 28 CGI-I ≤ 2
Pfizer [61]	340	Primary diagnosis of MDD without psychotic history	Desvelafaxine (25–50 mg), Fluoxetine (20 mg) placebo	1/3	12.7 (7–17)	54		8	Yes	NR	NR	No	NR	44	US, MX, CL	CGI-I ≤ 2
Pfizer [62]	363	Primary diagnosis of MDD without psychotic history	Desvelafaxine (20–35 mg), Desvelafaxine (25–50 mg), placebo	1/3	13 (7–17)	56.5		8	Yes	NR	NR	No	NR	42	US, MX,	CGI-I ≤ 2

*AE* United Arab Emirates, *AR* Argentina, *BDI* Beck depression inventory, *BE* Belgium, *C.P.* chance to receive placebo, *CA* Canada, *CDRS-R* Children’s Depression Rating Scale-revised, *CGI-I* Clinical Global Impression of Improvement Score, *CL* Chile, *com* combined (Emslie 2007a and b summarized these information), *CR* Costa Rica, *DE* Germany, *DSM-III* diagnostic and statistical manual of mental disorders, 3th edition, *DSM-III-R* diagnostic and statistical manual of mental disorders, 3th edition revised, *DSM-IV* diagnostic and statistical manual of mental disorders, 4th edition, *DSM-IV-TR* diagnostic and statistical manual of mental disorders, 4th edition, text revision, *EE* Estonia, *ES* Spain, *Eur.* Europe, *FI* Finland, *FR* France, *GB* United Kingdom, *IN* India, *IT* Italy, *Jp* Japan, *KADS* Kutcher Adolescent Depression Scale, *K-SADS* kiddie schedule for affective disorders and schizophrenia for school-age children, *MADRS* Montgomery-Âsberg Depression Rating Scale, *MDD* major depressive disorder, *MX* Mexico, *N* randomized sample size, *N sides* number of study sites, *NL* The Netherlands, *NR* not reported, *PBO* placebo, *PT* psychotherapy, *RADS* Reynolds Adolescent Depression Score, *RU* Russia, *SCL-58* Hopkins symptom checklist, *SK* Slovakia, *UA* Ukraine, *US* United States, *ZA* South Africa

*These study arms were not taken into account in our analysis

^Content of two randomized controlled trials, but data were summarized as one

### Search strategy, study selection and data extraction

In accordance with current guidelines [41], we searched for published trials in public databases including PubMed, Cochrane Library, Web of Science, PsychINFO, and for unpublished trials in clinical trial databases including Clinical Trial Registers of Australia (ANZCTR), China (CHiCTR), USA (ClinicalTrials.gov), Japan (UMIN-CTR), The Netherlands (Trial Register), the UN (ISRCTN), the World Health Organization (ICTRP), and the US Food &Drug Administration (FDA) for publicly accessible trial data up to September 5, 2017. In addition, references were identified from published articles and reviews [22, 24, 33, 36]. The search strategy for PubMed was “depressive disorder” (MESH) AND “antidepressive agents”, “second generation” (Pharmacological Action) AND “randomized controlled trial” (Publication Type) AND “Clinical Trial” (ptyp) OR “Randomized Controlled Trial” (ptyp) AND “human” (MeSH Terms) AND “child” (MeSH Terms) OR “adolescent” (MeSH Terms). We checked reference titles, abstracts and full texts for inclusion criteria. We identified and excluded duplicate records and collated multiple reports that relate to the same trial so that each trial rather than each report is the unit of interest in the review. Subsequently, two authors (MA, YP) independently extracted study characteristics and outcome data using a pre-defined extraction sheet with specific coding instructions. The extracted data contained methodological characteristics (study duration, sample size, study location, number of study sites, placebo run-in phase, publication year, assessment instruments, etc.), sample characteristics (mean age, proportion of females, baseline severity of depressive symptoms), intervention characteristics (antidepressive agent, permission to additionally receive psychotherapeutic treatment), and outcome data (primary and secondary outcomes). Risk of bias was assessed by two independent raters using the Cochrane risk of bias tool [41]. Any disagreements were resolved by discussion among at least two authors (RM, JA, YN, TP, and YP).

### Statistical analysis

Outcomes were response rates at the end of interventions as assessed with clinician-rating scales (primary), and with self-rating depression scales (secondary). Response according to the criteria laid out in the respective studies was either defined as a reduction of 50% on the depression scale from baseline to the end of treatment or as “much improved” or “very much improved” on the Clinical Global Impression Scale (see Table 1). Because response rates were not reported by two RCTs, we estimated them using the baseline mean, endpoint mean, and their standard deviations [63, 64]. For self-rated response analysis, response rates for all six studies had to be estimated [63, 64].

If more than one clinician-rating scale was used, we chose the one with the best psychometric properties as listed [65]: (1) The Children´s Depression Rating Scale Revised (CDRS-R), (2) the Hamilton Depression Rating Scale (HAMD), and (3) the Montgomery Asberg Depression Rating Scale (MADRS). In case of the application of both a clinician-rating scale for depression and the CGI-I, we preferred the clinician-rating scale. Similarly, in RCTs using more than one self-rating scale, we selected the following scales in the order as indicated: (1) Beck Depression Inventory (BDI), (2) Reynolds Adolescent Depression Scale (RADS), (3) Kutcher Adolescent Depression Scale-16 item (KADS), and (4) SCL-58 (Hopkins Symptom Checklist). The psychometric properties of the latter two measures had not been evaluated previously by Zhou et al. [65] and were thus placed at a low hierarchy.

We summarized the outcomes using odds with the corresponding 95% confidence intervals (CI). Odds were calculated using the intention-to-treat principle for all studies. The odds were log-transformed for all analyses and back transformed afterwards. Statistical analyses were performed according to the Cochrane Handbook for Systematic Reviews of Interventions [41]. We conducted random-effects meta-analyses using the restricted maximum likelihood estimator. The extent of statistical heterogeneity was tested for significance using Cochrane´s *Q* test and quantified by means of the *I*^2^ statistic [66]. We visually displayed the results as forest plots. To examine the impact of study duration and number of study sites on placebo response rates, we performed simple meta-regression analyses using the restricted maximum likelihood estimate method. Due to power considerations, we conducted meta-regression analyses only upon availability of at least ten studies for the respective analysis [41].

We also applied simple meta-regression analyses to explore all clinical and methodological effect modifiers that had previously been identified and seven additional modifiers (see final paragraph of the introduction). We then entered each modifier resulting in *p* value ≤ 0.05 in the respective simple meta-regression analysis into a multivariable meta-regression analysis including one or both of the two a priori hypothesized modifiers study duration and number of study sites in case they emerged as significant in the simple meta-regression analyses.

In total, the clinical effect modifiers were mean age, proportion of females, concomitant psychotherapy, and mean baseline severity of depression. To obtain comparable baseline severity despite the different measures which were used across trials, we computed three categories (“mild”, “moderate”, and “severe”) according to the cut-offs reported in the manuals.

The methodological modifiers were publication year, study duration, number of study sites, sample size, funding source (industry vs. independent), run-in phase, the chance to receive placebo and risk of bias. If RCTs did not report an explicit placebo run-in we assumed that it had not been performed. As noted in Table 2, studies with ≥ two (of six) applicable sources of bias were classified as high risk. Studies with one applicable source of bias and additional unclear bias control factors were classified as moderate, while studies with no more than one risk of bias and adequate control of all other five factors were classified as low risk of bias [22].

**Table 2:** Risk of bias in individual studies

	1	2	3	4	5	6	Global judgment
Atkinson et al. [44]	Yes	Yes	Yes	Yes	Yes	Yes	Low
Berard et al. [45]	Yes	Yes	Yes	No	Yes	Yes	Low
US Food and Drug Administration [37, , 46]	Unclear	Unclear	Unclear	Unclear	No	Unclear	Moderate
Lilly [47]	Unclear	Unclear	Unclear	Yes	Yes	No	Moderate
Emslie et al. [48]	Yes	Yes	Yes	Yes	No	Yes	Low
Emslie et al. [49]	Yes	Unclear	Yes	Yes	No	Yes	Moderate
US Food and Drug Administration [37, , 46]/Emslie et al. [38]	Unclear	Unclear	Unclear	Yes	No	Unclear	Moderate
Emslie et al. [50]	Yes	Unclear	Yes	Yes	Yes	Yes	Low
Emslie et al. [51]	Unclear	Unclear	Unclear	Yes	No	Yes	Moderate
Emslie et al. [51]	Unclear	Unclear	Unclear	Yes	No	Yes	Moderate
Emslie et al. [52]	Unclear	Unclear	Unclear	Yes	Yes	Yes	Moderate
Emslie et al. [53]	Yes	Yes	Yes	Yes	Yes	Yes	Low
Findling et al. [43]	Yes	Yes	Yes	Yes	Yes	Yes	Low
GSK [54]	Unclear	Unclear	Unclear	Yes	Yes	Yes	Moderate
Keller et al. [55]	Yes	Yes	Yes	Yes	Yes	No	Low
March et al. [56]	Yes	Unclear	Yes	Yes	Yes	Yes	Low
US Food and Drug Administration [39]/Cheung et al. [40]	Unclear	Unclear	Unclear	Unclear	No	Unclear	Moderate
US Food and Drug Administration [39]/Cheung et al. [40]	Unclear	Unclear	Unclear	Unclear	No	Unclear	Moderate
Von Knorring et al. [57]	Unclear	Unclear	Unclear	No	Yes	Yes	Moderate
Wagner et al. [57]	Yes	Yes	Yes	Yes	Yes	Yes	Low
Wagner et al. [59]	Unclear	Unclear	Unclear	Yes	Yes	Yes	Moderate
Wagner et al. [60]	Yes	Yes	Yes	Yes	Yes	Yes	Low
Pfizer [61]	Unclear	Unclear	Yes	Yes	Yes	Yes	Moderate
Pfizer [62]	Unclear	Unclear	Yes	Yes	Yes	Yes	Moderate

*1* allocation generation, *2* allocation concealment, *3* blinding of participant, personnel and outcome assessors, *4* incomplete data adequately addressed, *5* free of selective reporting, *6* free of other bias

To make our data comparable to the most recent adult data, we ran sensitivity analyses using the placebo response rates reported closest to week 8 [23].

Furthermore, a sensitivity analysis was performed within the studies utilizing a placebo-run phase, since these potentially impact the association of study duration and placebo repose.

All analyses were performed in the open source statistical environment R with the metafor package [67].

### Role of funding source

All authors are university employees, had full access to all data of the study and shared final responsibility for the decision to submit the results for publication. This study was neither funded by industry nor any specific grant.

## Results

### Included studies

**Fig. 1:**
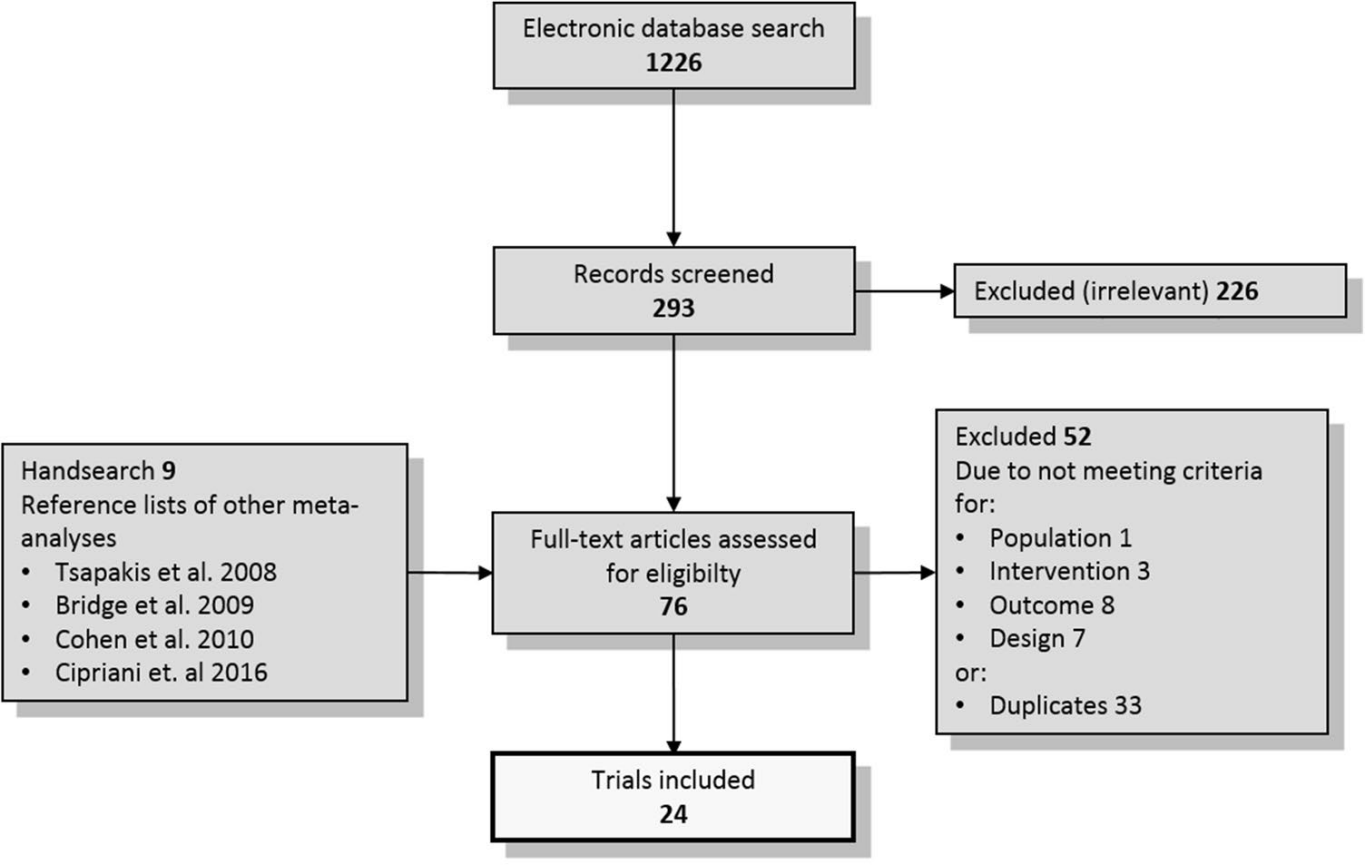
Study flow diagram

The literature search identified 24 placebo-controlled RCTs (a total of 2229 patients in the placebo arms) published between 1997 and 2017 with sample sizes ranging from 34 to 463 patients, as shown in the flow-chart (Fig. 1).

Fifteen of the 24 studies were conducted in one country only (thirteen in the United States, one each in Canada and Japan). Nine studies were conducted in more than one country, three thereof in Eurasian countries including Finland, France, Germany, Slovakia, Estonia, Russia, Ukraine, Belgium, Italy, Spain, United Kingdom, and The Netherlands and the remaining six studies in the United States, Mexico, Chile, South Africa, Canada, United Arab Emirates, Argentina, India, and Costa Rica.

The average age of the patients ranged from 12 to 16.5 years (with a weighted mean age of 12.5 years); the proportion of female patients ranged from 15 to 67%. Seventeen studies compared placebo with *one antidepressant in a single medication arm and seven studies compared placebo with different* antidepressants in at least two medication arms. Placebo was compared with citalopram (*k* = 2), desvenlafaxine (*k* = 2), duloxetine (*k* = 2), escitalopram (*k* = 2), fluoxetine (*k* = 8), nefazodone (*k* = 2), paroxetine (*k* = 4), mirtazapine (*k* = 2), sertraline (*k* = 1), and venlafaxine (*k* = 2). Most studies (*k* = 22 out of 24) were funded by industry. Six studies listed concomitant psychotherapy as an exclusion criterion, while the remaining studies (*k* = 18) did not prohibit concomitant psychotherapy explicitly but allowed it as part of TAU in both arms. The study duration ranged from 6 (*k* = 1) to 12 weeks (*k* = 3), with a peek at 8 weeks (*k* = 16). The baseline severity was mostly rated as moderate (*k* = 16). Ten studies conducted a placebo run-in phase as a screening prior to study start to exclude rapid placebo responders from study participation. The number of study sites ranged between one and 65, with a median of 26.5. Response criteria based on clinician-rating scales were reported in 22 studies. The detailed characteristics of each study are presented in Table 1.

### Risk of bias

The overall risk of bias was evaluated as “low” and “moderate” for eleven and thirteen studies, respectively. Knowledge of allocation was adequately prevented in twelve studies, whereas an adequate concealment of the allocation was reported in nine studies. The results of the methodological quality assessment are presented for all individual studies in Table 2.

### Overall placebo response rate

**Fig. 2:**
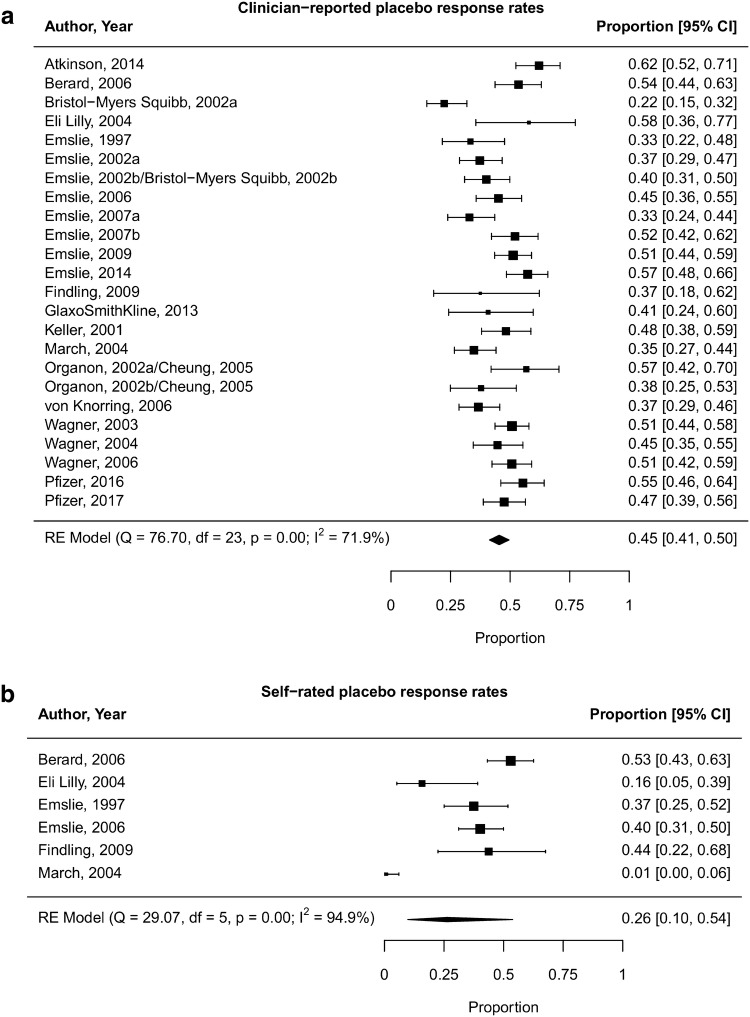
**a** Forest plot of clinician-rated response rates in the placebo arms at the end of intervention. **b** Forest plot of self-rated response rates in the placebo arms at the end of intervention

The clinician-rated placebo response rates (primary outcome) at the end of intervention ranged from 22 to 62% with a pooled response rate of 45% (95% CI 41–50%; *k* = 24; *n* = 2229; see Fig. 2a). The self-rated placebo response rates (secondary outcome) ranged from 0 to 68% with a pooled response rate of 26% (95% CI 10–54%; *k* = 6; *n* = 396; Fig. 2b). The *I* square values were high (71.9% for clinician-rating scales and 94.9% for self-rating scales) and indicated substantial heterogeneity in placebo response rates among the included trials.

### Modifiers of placebo response rates

**Fig. 3:**
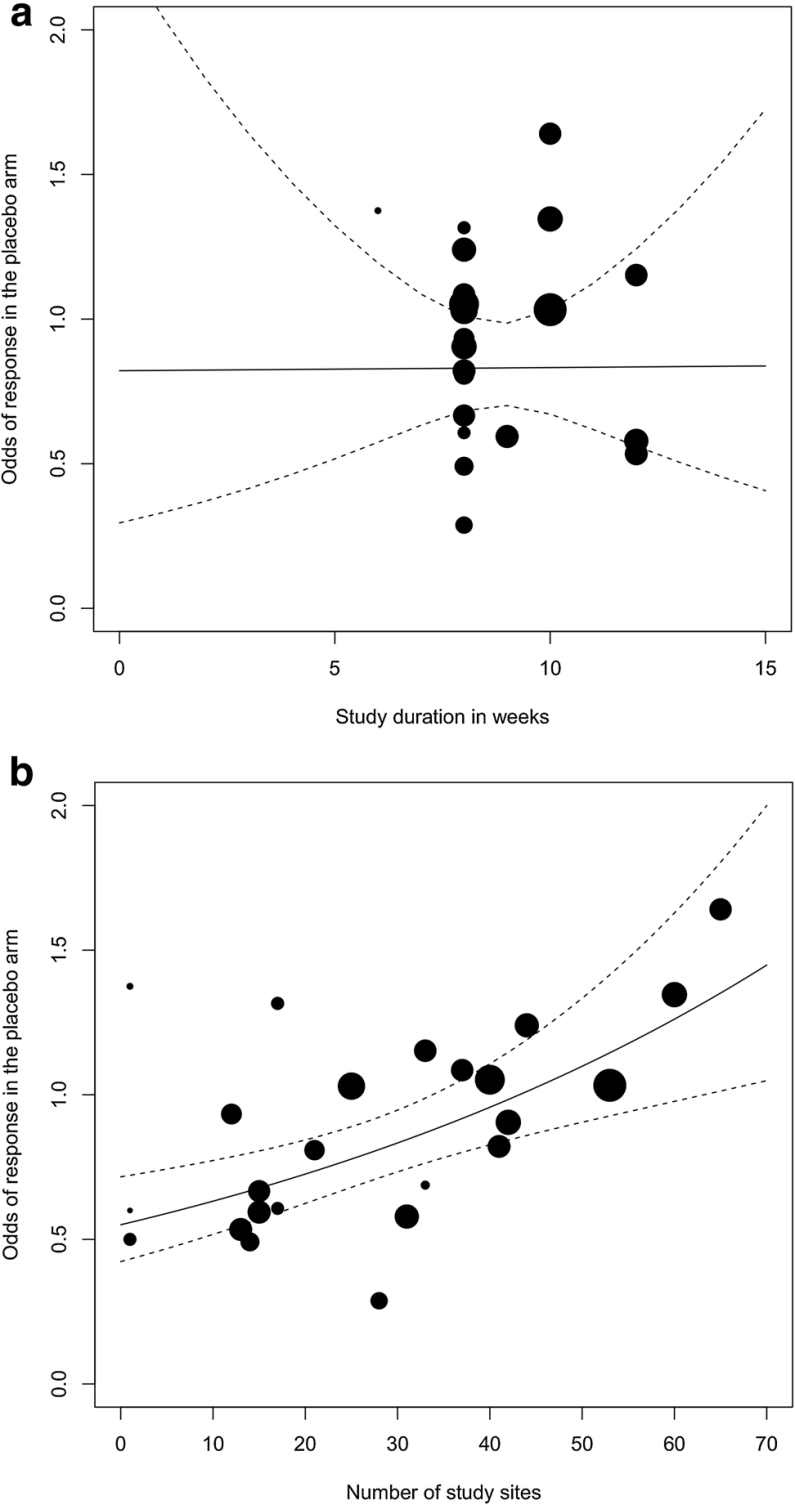
**a** Scatterplot showing the association between the odds of response in the placebo arm and the study duration in weeks. **b** Scatterplot showing the association between the odds of response in the placebo arm and the number of study sites

For clinician-rated placebo response rates at the end of intervention, simple meta-regression analyses revealed (Table 3) the number of study sites as a significant modifier (OR 1.01, 95% CI 1.01–1.02, *p* = 0.0003 for more study sites, *k* = 24; Fig. 3b) in accordance with the adult meta-regression of Furukawa et al. [23] and with the pediatric meta-regression of Bridge et al. [33]. Study duration on the other hand was not significant (OR 1.00, 95% CI 0.89–1.12, *p* = 0.984, *k* = 24; Fig. 3a).

**Table 3:** Modifiers of clinician-rated placebo response rates

Modifier/outcome	*N*	OR	95% CI	*P*
Confirmatory simple meta-regression analyses				
Study duration	24	1.00	0.89–1.12	0.982
Number of study sites	24	1.01	1.01–1.02	0.0003
Simple meta-regression analyses for additional modifiers				
Publication year	24	1.03	1.01–1.07	0.007
Baseline severity (ref. moderate)	18			
Severe		1.16	0.61–2.19	0.654
Run-in phase (ref. not performed)	24			
Performed		1.00	0.70–1.41	0.979
Co-psychotherapy (ref. not prohibited)	24			
Prohibited		0.98	0.67–1.42	0.896
Risk of bias (ref. low)	24			
Moderate		0.81	0.58–1.12	0.206
Funding source (ref. independent)	24			
Industry		1.67	0.93–3.00	0.086
Mean age	24	1.01	0.89–1.14	0.919
Proportion female	23	2.84	0.32–25.63	0.352
Sample size	24	1.00	1.00–1.00	0.178
Chance to receive placebo	24	0.30	0.04–2.08	0.221
Multivariable analysis (using all significant modifiers)				
Publication year	24	1.01	0.98–1.05	0.400
Number of study sites		1.01	1.00–1.02	0.042

*p p* value, *OR* odds ratio, *95% CI* 95% confidence interval, *N* number of studies

Among the explored clinical and methodological effect modifiers (Table 3), only publication year revealed a *p* value ≤ 0.05 (OR 1.03, 95% CI 1.01–1.07, nominal *p* = 0.007, *k* = 24). Moreover, industry funding revealed a trend for higher placebo response rates (OR 1.67, 95% CI 0.93–3.00, *p* = 0.086, *k* = 24).

We then entered publication year—the only modifier with a *p* value ≤ 0.05 in the explorative simple meta-regression analyses—into a multivariable meta-regression analysis together with number of study sites. Only the number of study sites remained a modifier (OR 1.01, 95% CI 1.00—1.02, nominal *p* = 0.042, *k* = 24; publication year: OR 1.01, 95% CI 0.98–1.0005, nominal *p* = 0.400, *k* = 24).

For our secondary outcome (self-rated placebo response rates at end of intervention) less than ten studies were available for a meta-regression analysis. In accordance with the Cochrane Handbook, we thus did not perform meta-regression analyses [41].

### Sensitivity analysis

Because Furukawa et al. [23] had based their meta-analysis on adult placebo response rates closest to week 8, we post hoc analyzed our data accordingly: the placebo response rates at “closest to week eight” (44%, 95% CI 38–49%) were comparable to those found at the end of intervention analysis (45%, 95% CI 41–50%). With regard to the two effect modifiers identified by Furukawa et al. [23], the sensitivity analysis of clinician-rated placebo response rates assessed closest to week 8 [23] again revealed the number of study sites (OR 1.02, 95% CI 1.01–1.03, nominal *p* = 0.002 for more study sites, *k* = 24) as a modifier of placebo response rates in children and adolescents. In contrast, the study duration did not result in a nominal *p* value ≤ 0.05 (OR 1.16, 95% CI 0.95–1.40, nominal *p* = 0.138, *k* = 24). On a descriptive level, an inverse U-shaped distribution was observed for the study duration (Fig. 3a). Thus, its impact on the placebo response increased until week 10 and decreased at week 12.

The funding source [OR 4.53, 95% CI: 1.96–10.45, nominal *p* = 0.0004 for industry sponsored (*k* = 22) vs. independent studies (*k* = 2)] and publication year (OR 1.04, 95% CI 1.01–1.08, nominal *p* = 0.025, *k* = 24) led to nominal *p* values ≤ 0.05 in the simple meta-regression analyses based on endpoint data closest to week 8. When publication year and funding source were entered together with the number of study sites, only funding source led to a *p* value ≤ 0.05 (OR 3.28, 95% CI 1.40–7.68, nominal *p* = 0.0061 for sponsored (*k* = 22) vs. independent studies (*k* = 2)).

Sensitivity analyses of the secondary outcome (self-rated placebo response rates) based on data closest to week 8 were not conducted as less than ten studies were available for each analysis.

The sensitivity analysis focusing on RCTs based on clinician ratings with a placebo run-in phase (*k* = 10, *n* = 865 patients in placebo arms) resulted in non-significant effects for the modifier study duration (last week reported: OR 1.05, 95% CI 0.88–1.22; closest to week 8: OR 0.75, 95% CI 0.52–1.08), while overall placebo response rates remained unchanged (last week reported: 45.5%, 95% CI 40.2–50.9%; closest to week 8: 44.6%, 95% CI 38.8–49.6%) as compared to the mean placebo response from all included trials.

## Discussion and outlook

This systematic review and meta-regression analysis represents the most comprehensive investigation of placebo response rates in double-blind RCTs comparing SNG-AD (SSRI, SNRI, SARI, and NaSSA) with placebo in the acute treatment of children and adolescents with a primary diagnosis of MDD. Moreover, we investigated a variety of potential modifiers of placebo response rates including one that to our knowledge has not yet been investigated in children and adolescents (concomitant psychotherapy). This enables us to compare our results on children and adolescents with those of Furukawa and colleagues on adults [23]. In addition, this is the first meta-analytical investigation of self-rated placebo response rates in children and adolescents with MDD.

### Placebo response rates

We found a pooled mean clinician-rated placebo response rate of 45% with a 95% confidence interval ranging from 41 to 50% (Fig. 2a). By that, placebo responses in children and adolescents are higher than the reported mean response rate of 36% with 95% confidence intervals ranging from 35 to 37% for comparable studies performed in adults [23].

Our results are comparable with previous systematic reviews and meta-analyses encompassing children and adolescents with MDD [24, 33, 36]. Cohen and colleagues (*k* = 23 for MDD) and Tsapakis and colleagues (*k* = 30) included both SNG-AD and tricyclic antidepressants and found a mean placebo response rate of 49.6% (95% CI 17–90%) and median response rate of 49.2% (95% CI 35.7–59.1%), respectively [24, 36]. Bridge [33] and colleagues (*k* = 12) included SNG-AD only and found a mean placebo response rate of 46% ranging from 33 to 57%. Bridge et al. 2007 [34] (*k* = 15 for MDD) aimed to assess the efficacy and safety of SNG-AD for MDD, but also for obsessive–compulsive disorder (OCD) and non-OCD anxiety disorder and found a pooled placebo response rate of 50% (95% CI 47–53%) for MDD. Locher et al. [35] (*k* = 17 for MDD) also reported placebo response rates, but defined them as the mean change scores of preanalyses vs. postanalyses in the placebo groups. Thus, they operationalized placebo response as a continuous outcome, while we operationalized placebo response as a dichotomous outcome. Both the continuous and the dichotomous outcomes have advantages and disadvantages. Dichotomous outcomes on the one hand indicate how many patients profited from the intervention and are relatively easy to understand for clinicians and patients. On the other hand, dichotomizing outcomes results in a loss of information, reduced power and artificial boundaries [68, 69].

For clinicians and patients, however, Cohen’s d is difficult to understand and does not indicate how many patients benefited from the treatment. Dichotomous outcomes, on the other hand, are relatively easy to understand for clinicians and consumers, but there is no consensus about the criteria for these efficacy standards [69] and the statistical power of detecting significant differences is smaller than the power of Cohen’s d.

The overlap of the 24 included trials of our study with each of these reviews is as follows: thirteen of the 23 MDD trials analyzed by Cohen et al. [24], fourteen of the 30 trials analyzed by Tsapakis et al. [36], and twelve of the twelve studies analyzed by Bridge et al. [33], all of the 15 studies analyzed by Bridge et al. [34] and all of the 17 studies analyzed by Locher et al. [35]. With regard to the number of included studies with only SNG-AD (*k* = 24), our current meta-analysis is the most comprehensive.

We calculated a pooled mean placebo response rate of 26% with a broader range of the 95% CI from 10 to 54% for self-rated response; however, this analysis was based on a substantially lower number of pediatric trials (*k* = 6; *n* = 396 patients); the large 95% confidence interval observed for self-rated placebo response rates reflects the low power and overlaps with that observed for clinician-rated placebo response rates. The lower mean response rate is in line with results from adult studies according to which the proportion of adult placebo responders based on self-ratings was only one-third of that observed for clinician ratings [70]. Taken together, we hypothesize that the placebo response rates based on self-ratings may prove to be lower than those based on clinician ratings across the whole age range; however, future studies are necessary to prove this hypothesis due to the low power of pediatric studies. Self- and clinician-rated depression severity is known to be only modestly correlated [71–73]; younger age, depressive subtype, and higher educational attainment have been shown to account for higher BDI scores relative to clinician ratings (HAMD) [73]. With respect to remission, only about half of the patients assessed as remitted by the HAMD score in a more recent study, considered themselves as being remitted [74]. Other pediatric meta-analyses had not analyzed self-rated placebo response rates.

Placebo responses are considered to be driven by implicit or explicit treatment expectations induced by verbal information or prior experience (learning) and have been associated with distinct neurobiological mechanisms [25]. These mechanisms are being also studied in the field of depression; first studies point towards the relevance of, e.g., the prefrontal cortex, anterior cingulate, premotor, parietal, posterior insula, and posterior cingulate [75]. The prefrontal cortex, for example, undergoes significant maturation throughout childhood and puberty which may interfere with one’s ability to generate and maintain placebo responses [29]. Both psychological and neurobiological factors relevant to the development and maintenance of treatment expectation are subject to significant neurodevelopmental changes across the lifespan [29]. For instance, a study investigating placebo analgesic responses in children and adolescents [76], has provided evidence that a learning mechanism plays a stronger role in driving placebo analgesic effects in children and adolescents than in adults. It has further been hypothesized that associative learning mechanisms including the observation of treatment effects in others (“placebo by proxy”) may play a crucial role in placebo responses in children and adolescents [77].

Suggestibility differs between individuals and develops over time. It has been demonstrated that suggestibility in children around 12 years reaches 80% while 15% of adults are highly suggestible. This factor could also play a role in the high placebo response in children and adolescents [29]).

Unfortunately, the granularity of the published details does not allow an in depth evaluation of potential causes.

Tsapakis et al. [36] reasoned that study samples of juvenile patients with MDD might be even more heterogeneous than adult study samples. They argued that juvenile trials may include higher proportions of mildly depressed patients without prior experience to hospitalized treatments who may speculatively improve spontaneously, adding therewith to the high effect of placebo treatments observed for young patients. The hypothesis of Tsapakis [36] of an elevated spontaneous improvement rate in youths as compared to adults is intriguing, but hard to prove, since studies on the “effect” of “no-treatment” are scarce. Since a placebo response is not equal to spontaneous improvement, studies which compare the placebo arm with a no-treatment arm are warranted to extract the “real” placebo effect, because clinical changes might occur due to the “placebo intervention”, but also due to regression towards the mean, or even spontaneous remission [35], or finally a combination of these effects [78]. To unravel the “true” placebo effect it will be necessary to consider the difference between the placebo response rate upon a placebo intervention and the spontaneous improvement rate in untreated patients. Gold standard designs [25, 79] would thus consider no-treatment arms [80], which, however, entail ethical issues that would need to be addressed.

One potential way to unmask the contribution of the placebo pill per se is to omit the concealment character and inform the patient that he/she will receive an inert substance within trials applying the so-called open-label placebo regimen [29, 81]. To our knowledge, there is only one such open-label pilot placebo study for MDD in adults which compared placebo with no treatment [82]. Unfortunately, there is not yet any open-label placebo study for children and adolescents with MDD. We cautiously speculate that a medium or even high effect size for this age group might apply. Accordingly, we support future open-label placebo studies in children and adolescents with MDD, or other study types that make clinical use of the documented high placebo responses in this age group.

Tsapakis et al. [36] suggested that the relatively higher placebo response rates in children and adolescents represent the main reason for the so-called “failed” antidepressant trials for this age range. In contrast, the *verum* response rates in adolescents were observed to be very similar to those in adults. The authors specifically noted that the similar *verum* response is in accordance with the similar pharmacokinetics in adolescence and adulthood [83]. In conclusion, the higher placebo response in the pediatric age range results in a difference that is too small between *verum* and placebo effects, which in turn, frequently leads to a failure to prove superiority of the studied *verum* [84]. Potential other reasons for failure might depend on the challenging differential diagnosis between ‘true’ MDD in young patients and other behavioral and emotional disorders or a reduced mean length of a depressive episode in children and adolescents.

### Modifiers of placebo response rates

The most recent and comprehensive meta-analysis of antidepressant trials in adults with a focus on placebo response rates [23] identified study duration and number of study sites to be consistently and independently associated with placebo response. Our pediatric meta-regression analysis based on a substantially smaller number of studies (*k* = 24 vs. *k* = 252) was not able to identify study duration; however, we too [33] identified the modifying influence of number of study sites (OR = 1.01, 95% CI 1.01–1.02, *p* = 0.0003 for more study sites, *k* = 24). This association remained stable when controlling for publication year (OR = 1.01, 95% CI 1.00–1.02, *p* = 0.042 for more study sites, *k* = 24), which in analogy to adult analyses [23, 28] and pediatric analyses [33] emerged as a modifier in our explorative simple meta-regression analysis.

Based on results obtained in adults [23] one of our two tested hypotheses pertaining to modifiers was that the placebo response is more pronounced in studies of a longer duration. However, this hypothesis was not supported (end of intervention data: OR 1.00, 95% CI 0.89–1.12, *p* = 0.984, *k* = 24; closest to week 8 data: OR 1.16, 95% CI 0.95–1.40, *p* = 0.138, *k* = 24). Overall, our results are in accordance with those published by Bridge et al. [33], who found no significant correlation between the proportion of placebo responders and the duration of the treatment period. This might be speculatively due to the considerably lower number of RCTs and therewith lower number of individual cases in the investigated trials of children and adolescents. In adults, the significant relative risk (RR) of 1.03 (95% CI 1.01–1.05) for 1 additional week in trial length using data closest to week 8 was observed [23]. It appears of interest to determine if as in our analysis the odds ratio in adults also decreases upon use of end of study data.

We additionally investigated other clinical and methodological modifiers of placebo response in an explorative manner. We had selected these modifiers, because they had been identified in at least one of the six previous meta-analyses with pediatric studies as modifier of placebo response [24, 33] or because of theoretical considerations or reviewer suggestions. Only publication year predicted placebo response rate in a simple meta-regression analysis, but this association was no longer apparent when entered in a multivariable model including number of study sites. This finding is in accordance with the findings of the most recent adult study [23] and seemingly reflects the average increase of the number of study sites per study over the years.

In accordance with our results which showed that age is not a modifier of the placebo response in pediatric MDD patients, Bridge et al. [33], too, did not find a significant difference in placebo response rates between adolescents (44.5%) and children (49.6%). An overall age effect for placebo response rates was not identified in recent adult studies [23, 28]. However, a meta-analysis in older adults (mean age > 60, *k* = 6 studies) showed a significantly higher placebo response rate and a relatively lower medication response [85]. Potentially, future RCTs should include both adolescents and adults to overcome the current dichotomy; however, a single clinician-rating diagnostic tool would be required that covers the respective age range.

Regarding baseline severity of MDD, the drug-placebo difference was significant for adult patients in the upper range of severe depression according to Kirsch et al. [86], whereas a recent analysis of patient-level data of 34 RCTs did not support this finding [87]. Potentially due to the small number of severely depressed patients in our analysis (only two trials reported a mean score in the “severely depressed” range at baseline), the modifier baseline severity was non-significant (OR 1.16, 95% CI 0.61–2.19; *p* = 0.654, *k* = 18). However, recent meta-analyses in children and adolescents with MDD focusing on modifiers of placebo response [24, 33] showed that youths with severe baseline depression presented a smaller association with placebo response, if symptoms were measured with the CGI-I. However, this association became insignificant in multivariable regression analysis if controlled for number of study sites [33].

Considering end of study data (OR 1.67, 95% CI 0.93–3.00; *p* = 0.086, *k* = 24), funding source showed a trend for a modifying effect. Finally, in our close to week 8 sensitivity analysis, the funding source showed to be a modifier (OR 4.53, 95% CI 1.96–10.45; nominal *p* = 0.0004, *k* = 24). Due to the small number (*k* = 2 out of 24) of non-industry sponsored studies analyzed by us, the results need to be interpreted with caution and cannot be generalized.

Finally, the modifier sample size which was found to be associated with placebo response in one pediatric study [correlation: *r* = 0.71 (0.23–0.91; *t* = 3.18; *p* ≤ 0.01) *k* = 12] [33] did not show up in our explorative analysis (OR 1.00, 95% CI 1.00–1.00; nominal *p* = 0.178; *k* = 24). However, because of the relatively small number of studies and therewith low statistical power, null findings need to be interpreted with caution.

It is important to bear in mind that our analysis focused on placebo response only. Thus, the important and continuing scientific debate about the efficacy of antidepressants in general [86, 88] does not represent the focus of this meta-analysis. Based on our results we nevertheless suggest that the larger clinician-rated placebo response rates confirmed in our analysis seem to be age dependent [77] and diminish the *verum* effect of antidepressants in RCTs in children and adolescents [22] more than in adult trials [32].

Limitations: We cannot rule out that our search failed to identify single studies, especially if published in another language than English or if published not at all in the public domain or reported in clinical trial databases. However, we did not limit our search to articles in English language. We included only trials that studied a priori defined SNG-AD. However, this was decided intentionally by all authors in light of the current best practice in child and adolescent psychiatry [18–20]. We faced a substantial heterogeneity as revealed by high I-squared values. The pooled placebo response rates should therefore be interpreted with due caution. Further limitations result from the methodology of meta-regression analyses, which may produce false-positive associations that reflect no true associations but are caused by chance alone [89]. However, our results for children and adolescents with respect to modifiers were similar to those obtained in a similar adult study conducted by Furukawa et al. [23], thus overall supporting the validity of the obtained results. Moreover, our sensitivity analyses revealed similar modifiers of placebo response in the simple meta-regression analyses, but some discrepancies appeared in the multivariable multi-regression-analysis as detailed above. As meta-regression analyses are based on aggregated data of primary studies, only conclusions on a study level and not on an individual level can be drawn [87, 90]. Meta-regression analyses on individual data may therefore lead to different results. In comparison to the recent study of Furukawa et al. [23], the number of included pediatric studies for the current analysis was approximately only one tenth of that of the adult study (*k* = 24 vs. *k* = 252). As such, the low power of our study needs to be pointed out as a limitation. Accordingly, our negative findings must be interpreted with caution. We conducted all analyses in accordance with the Cochrane Handbook for Systematic Reviews of Interventions and only performed simple meta-regression analyses when at least ten studies were available. Due to power considerations, we additionally conducted only simple meta-regression analyses in the first step. The reduced statistical power of our study may have precluded the detection of small effects. Moreover, we did not adjust for the testing of both the primary and secondary outcomes; furthermore, we did not correct the *p* values for our hypothesis driven analysis of the two modifiers number of study sites and study duration. Because of the post hoc character of the explorative analysis of previously identified modifiers and our sensitivity analyses, we reported nominal *p* values only.

In conclusion, the pooled placebo response rates in children and adolescents are higher than the rates observed in adult patients with MDD. This represents a major challenge for future clinical trials of antidepressants in this age group. Clinician-rating resulted in higher placebo response rates than patient based data. Just as in the adult literature, this effect might trigger a discussion of the differential validity of these types of outcome assessments. Furthermore, only the number of study sites was a consistent modifier, with a small effect for stronger placebo responses in larger multisite trials. Since large scale multisite studies are state-of-the art, we believe that this result supports our claim for a stronger consideration of ways to make use of the strong antidepressant effects of placebos in clinical practice such as open-label treatments. Importantly, placebo responses in children and adolescents were stable irrespective of study duration, age, baseline depression severity, or risk of bias. While pharmaceutical industry sponsored studies are usually designed to minimize the impact of placebo on study results (e.g., by facilitating placebo-run in phases—interestingly, run-in phase was not identified as a modifier in our study) to meet the study goals, practicing clinicians might rather want to facilitate placebo effects as an addition to the *verum* effect to maximize benefits [25]. Future trials and meta-analyses should include both medium and longer term follow-ups to further analyze the duration and stability of the strong placebo response in children and adolescents with MDD.

## Electronic supplementary material

Below is the link to the electronic supplementary material.

Supplementary material 1 (PDF 106 kb)
