# Supplementary material for: Placebo response rates and potential modifiers in double-blind randomized controlled trials of second and newer generation antidepressants for major depressive disorder in children and adolescents: a systematic review and meta-regression analysis
Source: Eur Child Adolesc Psychiatry. 2018 Dec 8;29(3):253–73. doi: 10.1007/s00787-018-1244-7 (PMC7056684; doi:10.1007/s00787-018-1244-7)
Supplement: Supplementary file 1 — Supplementary material 1 (PDF 106 kb) [file 787_2018_1244_MOESM1_ESM.pdf]

Supplementary appendix to the manuscript

Study	pub_year	dur_depres_epis_at intake (m)	Duration	mean age	prop_female	sample size	chance_plac	drug class	drug	fund_ source	Sum_risk_of_Bias	Cut_off_basesever_clin	Cut_off_basesever_self	resp_measure_scale	resp_def	CoPsychotherapy	studysites	Nstudy_sites	runinphase_imp	Clin_Nresp_pb	Self_Nresp_pb	NITT_pb
Atkinson, 2014	2014	NR	10	13,2	0,52	337	0,33	SSRI, SSNRI	Fluoxetine, Duloxetine	industry sponsored	low	moderate		CDRS	reduction ≥ 50%	PT not prohibited	multi-center	65	no run-in phase	64		103
Berard, 2006	2006	NR	12	15,6	0,67	286	0,50	SSRI	Paroxetine	industry sponsored	low	moderate	moderate	MADRS	reduction ≥ 50%	PT prohibited	multi-center	33	run-in phase	53	52	99
Bristol-Myers Squibb, 2002a	2002	NR	8	12,0	0,50	284	0,33	DAS, DSA	Nefazodone	industry sponsored	moderate	moderate		#NULL!	#NULL!	PT not prohibited	multi-center	28	no run-in phase	21		94
Eli Lilly, 2004	2004	NR	6	15,6	0,55	40	0,50	SSRI	Fluoxetine	industry sponsored	moderate	moderate	moderate	#NULL!	#NULL!	PT not prohibited	single-center	1	run-in phase	11	3	19
Emslie, 1997	1997	3,4	8	12,4	0,46	96	0,50	SSRI	Fluoxetine	independent	low	moderate	mild	CGI-I	score of 1 or 2	PT not prohibited	single-center	1	run-in phase	16	18	48
Emslie, 2002a	2002	15,3	9	12,7	0,49	219	0,50	SSRI	Fluoxetine	industry sponsored	moderate	moderate		CDRS	reduction ≥ 50%	PT prohibited	multi-center	15	run-in phase	41		110
Emslie, 2002b/Bristol-Myers Squibb, 2002b	2002	NR	8	15,0	0,60	206	0,50	DSA	Nefazodone	industry sponsored	moderate			CGI-I	score of 1 or 2	PT not prohibited	multi-center	15	no run-in phase	40		100
Emslie, 2006	2006	24,9	8	12,0	0,47	206	0,50	SSRI	Paroxetine	industry sponsored	low	severe	moderate	CGI-I	score of 1 or 2	PT prohibited	multi-center	41	no run-in phase	46	41	102
Emslie, 2007a	2007	23,1	8	12,2	0,50	165	0,50	SSNRI	Venlafaxine	industry sponsored	moderate	moderate		HAMD	reduction ≥ 50%	PT not prohibited	multi-center	14	run-in phase	28		85
Emslie, 2007b	2007	23,1	8	12,2	0,42	201	0,50	SSNRI	Venlafaxine	industry sponsored	moderate	moderate		HAMD	reduction ≥ 50%	PT not prohibited	multi-center	37	run-in phase	51		98
Emslie, 2009	2009	16,5	8	14,6	0,59	316	0,50	SSRI	Escitalopram	industry sponsored	moderate	moderate		CDRS	reduction ≥ 50%	PT not prohibited	multi-center	40	run-in phase	81		158
Emslie, 2014	2014	NR	10	13,0	0,51	463	0,25	SSRI, SSNRI, SSNRI	Fluoxetine, Duloxetine	industry sponsored	low	moderate		CDRS	reduction ≥ 50%	PT not prohibited	multi-center	60	no run-in phase	70		122
Findling, 2009	2009	61,2	8	16,5	0,15	34	0,50	SSRI	Fluoxetine	industry sponsored	low	moderate	mild	CGI-I	score of 1 or 2	PT not prohibited	single-center	1	no run-in phase	6	7	16
GlaxoSmithKline, 2013	2013	NR	8	14,6	0,61	56	0,50	SSRI	Paroxetine	industry sponsored	moderate			CGI-I	score of 1 or 2	PT not prohibited	multi-center	33	run-in phase	11		27
Keller, 2001	2001	13,0	8	14,9	0,62	275	0,33	SSRI	Paroxetine	industry sponsored	low	moderate		CGI-I	score of 1 or 2	PT prohibited	multi-center	12	no run-in phase	42		87
March, 2004	2004	8,9 (median)	12	14,6	0,54	439	0,50	SSRI	Fluoxetine	independent	low	moderate	moderate	CGI-I	score of 1 or 2	PT prohibited	multi-center	13	no run-in phase	39	1	112
Organon, 2002a/Cheung, 2005	2002	NR	8	12,3	0,51	126	0,50	NaSSA	Remeron	industry sponsored	moderate			CGI-I	score of 1 or 2	PT not prohibited	multi-center	17	no run-in phase	25		44
Organon, 2002b/Cheung, 2005	2002	NR	8	12,0	0,53	133	0,50	NaSSA	Remeron	industry sponsored	moderate			CGI-I	score of 1 or 2	PT not prohibited	multi-center	17	no run-in phase	17		45
von Knorring, 2006	2006	NR	12	16,0		244	0,50	SSRI	Citalopram	industry sponsored	moderate	moderate		MADRS	reduction ≥ 50%	PT not prohibited	multi-center	31	no run-in phase	44		120
Wagner, 2003	2003	NR	10	12,0	0,51	376	0,50	SSRI	Sertraline	industry sponsored	low	severe		CGI-I	score of 1 or 2	PT prohibited	multi-center	53	no run-in phase	95		187
Wagner, 2004	2004	20,0	8	12,1	0,53	178	0,50	SSRI	Citalopram	industry sponsored	moderate	moderate		CGI-I	score of 1 or 2	PT not prohibited	multi-center	21	run-in phase	38		85
Wagner, 2006	2006	15,6	8	12,3	0,52	268	0,50	SSRI	Escitalopram	industry sponsored	low	moderate		CGI-I	score of 1 or 2	PT not prohibited	multi-center	25	run-in phase	69		136
Pfizer, 2016	2016	NR	8	12,7	0,54	340	0,33	SNRI, SSRI	Desvenlafaxine, Fluoxetine	industry sponsored	moderate			CGI-I	score of 1 or 2	PT not prohibited	multi-center	44	no run-in phase	62		112
Pfizer, 2017	2017	NR	8	13,0	0,57	363	0,33	SNRI	Desvenlafaxine, high- and low dose	industry sponsored	moderate			CGI-I	score of 1 or 2	PT not prohibited	multi-center	42	no run-in phase	57		120

pub_year=publication year; prop_female=proportion female; chance_plac=chance to receive placebo; fund_source=funding source; Cut_off_basesever_clin=Cut-off baseline severity clinician-rated; Cut_of_basesever_self= Cut-off baseline severity self-rated; resp_measure_scale=response measurement scale; resp_def=response definition; CoPsychotherapy=concomitant psychotherapy; Nstudy sites=number of study sites; runinphase_imp.=run-in phase imputed; Clin_Nresp_pb=number of placebo responder clinician-rated; Self_Nresp_pb=number of placebo responder self-rated; NITT_pb=number of intention-to-treat population; #NULL!/NR=not reported
